# Exploring Sibling Relationship Quality among Latinx Siblings: A Systematic Review

**DOI:** 10.3390/bs14070624

**Published:** 2024-07-22

**Authors:** Megan R. Holmes, Kari A. O’Donnell, Kristina Lovato, Laurie Kramer, Amy E. Korsch-Williams, Allison E. Herceg, Sylvia O. Stephens

**Affiliations:** 1 Center on Trauma and Adversity, Mandel School of Applied Social Sciences, Case Western Reserve University, Cleveland, OH 44106, USA; kao40@case.edu (K.A.O.); axk130@case.edu (A.E.K.-W.); aeh180@case.edu (A.E.H.); sos22@case.edu (S.O.S.); 2 School of Social Welfare, University of California, Berkeley, CA 94720, USA; kristina.lovato@berkeley.edu; 3 Department of Applied Psychology, Northeastern University, Boston, MA 02115, USA; l.kramer@northeastern.edu

**Keywords:** sibling relationship quality, Latinx families, cultural factors, familism, simpatía, gender dynamics, systematic review, family dynamics, cultural values

## Abstract

This systematic review addresses the gap in the literature regarding sibling relationship quality among Latinx families, a topic that has not been comprehensively examined to date. This study aimed to synthesize current research on sibling relationship quality in Latinx families, focusing on the influence of cultural factors, identifying key variables associated with sibling relationship quality, and evaluating the methodological approaches used. This paper is a systematic review based on a recently published evidence and gap map (EGM) that identified and visually presented all published studies investigating sibling relationship quality. Additional inclusion criteria were applied to select articles that specifically examined sibling relationship quality in the United States samples with at least 50% Latinx participants. The review included 12 articles representing 6 distinct studies, revealing significant findings on the roles of familism, simpatía, and gender in shaping sibling dynamics. Results indicated that cultural values such as familism and simpatía positively influence sibling intimacy and warmth, while gender dynamics further moderate these relationships. Methodologically, the articles employed longitudinal and cross-sectional designs, utilizing various quantitative measures. The findings underscore the importance of culturally sensitive approaches in studying sibling relationships and highlight the need for further research to explore these dynamics in diverse Latinx subgroups.

## 1. Introduction

As one of the longest-standing relationships across the life course, siblings have major influences on human development and behavior [1,2,3,4,5,6]. Siblings learn from each other during everyday moments of play and family activities, which provide opportunities for them to acquire important social, emotional, and behavioral skills [5,7]. In the United States, most children (77–82%) grow up with at least one sibling [8]. Existing research on sibling relationships has primarily focused on majority white, middle-to-upper class, and/or two-parent intact family samples [9]. While this research has provided valuable insights, it also has lacked cultural specificity. By systematically reviewing and synthesizing existing studies on Latinx families, this paper provides a comprehensive understanding of how cultural factors influence sibling dynamics, identifies key variables associated with positive sibling relationships, and evaluates the methodological approaches used in existing studies. This review fills a critical gap in the literature by providing a comprehensive synthesis of the unique cultural factors influencing sibling relationships in Latinx families.

### 1.1. Sibling Relationship Quality

Although some studies have indicated that sibling relationships can be a context of conflict and aggression [10,11], they can also contain some of the closest emotional bonds a person has throughout life [12,13]. Warm, nurturing, and close sibling relationships significantly contribute to children’s social skills with peers, their ability to resolve conflicts constructively, and their social and emotional comprehension [14,15,16,17]. Sibling relationships serve as a critical foundation for later social development, influencing both peer and romantic relationships [14,15,16,17]. This cascading influence on other key relationships throughout life positions sibling relationships as a potentially transformative target for intervention.

### 1.2. Latinx Families

Despite nearly 40 years of sibling relationship quality research, most existing sibling research has utilized majority white, middle to upper class, and/or two-parent intact family samples [9]. This limits our understanding of how the sibling relationship is experienced for children in diverse family environments. Notably, there continues to be substantial growth within Latinx communities in the United States [18], which now constitute the largest racial and ethnic minority as well as immigrant group [19]. Latinx communities are diverse and heterogeneous and trace their family heritage across Latin America. As of 2018, Spanish-speaking Latinx communities comprised approximately 18% of the U.S. population, totaling around 60 million individuals [20]. According to the National Research Center on Hispanic Families [21], which analyzed data from the 2019 American Community Survey, 66 percent of Hispanic children (12.2 million) in the U.S. have Mexican origins. Additionally, around 9 percent (1.8 million) have Puerto Rican origins, nearly 4 percent (695,000) are from El Salvador, and about 3 percent have origins in the Dominican Republic (584,000), Guatemala (547,000), and Cuba (481,000). Over 37 million Latinx individuals, or 34.8% of those aged five and above, use Spanish as their primary language at home, establishing Spanish as the most prevalent non-English language spoken in the country [20]. The limited work that has been conducted with diverse sibling populations suggests that sibling relationship quality may vary across racial, ethnic, and/or cultural groups [22,23]. Focusing on siblings with diverse socioeconomic identities in research will build a more comprehensive understanding of the factors that influence sibling relationship quality in different family contexts. 

Sibling relationships have been recognized as a central aspect of family life in Latinx culture. Approximately 77% of Latinx children have one or more siblings and spend more time engaged in activities with siblings than with peers, extended family members, or parents [24,25,26]. Familism/familismo is a construct that characterizes Mexican and Latinx families’ lives by prioritizing family support, responsibilities, interdependence, and involvement [27,28]. Familism values are associated with the quality of sibling relationships [29] and youth engagement in prosocial behaviors [30]. Respect for family members, the importance of elders, family hierarchy, and the role of gender are also frequently described as important factors in Latinx culture [28,31]. Simpatía is a key value in Latinx cultures that emphasizes smooth, harmonious social interactions and conflict avoidance [32]. It involves being agreeable, polite, and respectful to maintain social harmony. This value promotes warmth, friendliness, and a positive atmosphere in relationships. Simpatía can influence family dynamics and sibling relationships by fostering a supportive and nurturing environment where individuals prioritize the feelings and well-being of others. Gender roles, such as machismo and marianismo, may influence sibling dynamics by shaping expectations and behaviors; males are often seen as protectors and providers, while females are viewed as caregivers and nurturers [33,34]. These traditional gender roles may affect how siblings interact, support each other, or resolve conflicts. Cultural ideals emphasizing collectivism, cohesion, affiliation, and relatedness have been depicted as important influences on parenting practices [35,36,37]. Given the unique cultural context of Latinx families, it is essential to understand how these factors shape sibling relationship quality.

### 1.3. Current Study

To date, no systematic review, to the best of our knowledge, has been published specifically examining sibling relationship quality among Latinx families. However, Updegraff et al. [25] wrote a book chapter that focused on understanding children’s and adolescents’ sibling relationships across different cultural contexts. While this chapter summarized how cultural norms and values influence sibling roles and dynamics through an ecological perspective, it was not a systematic review and was published over ten years ago. Therefore, this study aims to systematically review and synthesize existing sibling relationship quality studies among Latinx families, with particular emphasis on understanding how cultural factors influence sibling dynamics, identifying key variables associated with positive sibling relationships, and evaluating the methodological approaches used in existing research.

## 2. Methods

**Figure 1:**
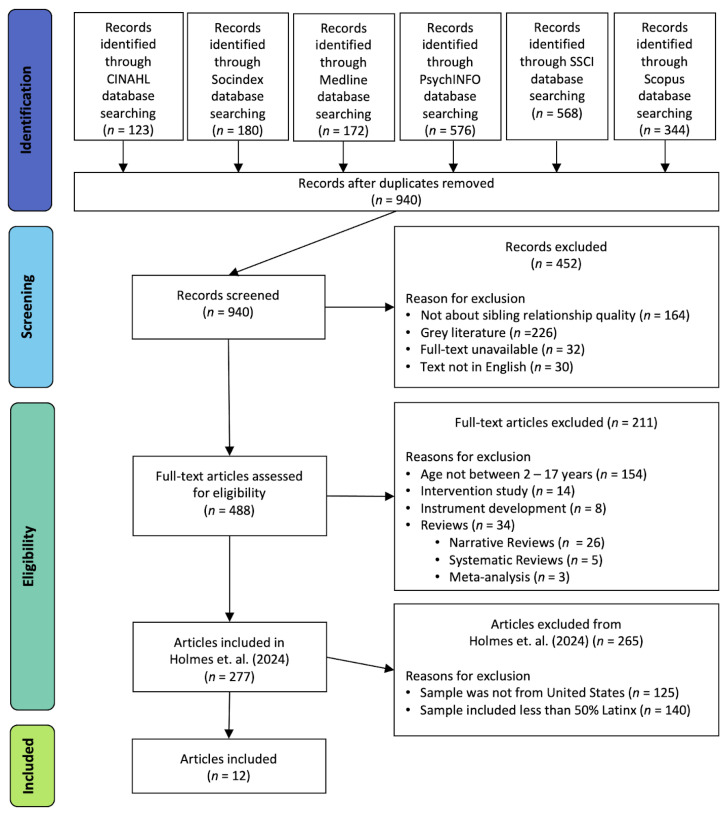
Flow of information through each phase of the systematic review. SSCI, Social Science Citation Index [9].

This systematic review aims to synthesize existing research on sibling relationship quality among Latinx families, focusing on cultural influences, associated factors, and methodological approaches. This review is based on the Holmes et al. [9] evidence and gap map (EGM) that identified and visually presented all published studies investigating sibling relationship quality. As shown in Figure 1, the study followed procedures outlined by Saran and White [38] from the Campbell Collaboration, involving comprehensive and systematic searches and coding of evidence. This review follows the Preferred Reporting Items for Systematic Reviews and Meta-Analyses (PRISMA) guidelines.

Briefly, six electronic bibliographic databases were searched: CINAHL, Medline, PsycINFO, Social Science Citation Index, Scopus, and Socindex. The key search terms included “(SU sibling* or ‘sibling relation*’ or brother* or sister*) AND (relationship N3 quality) AND (child* or youth or adolescent* or toddler* or infant*).” Inclusion criteria for the studies were as follows: examining sibling relationship quality, including children between ages 2 and 17, and being published in a peer-reviewed journal. Exclusion criteria included studies on the child response to the birth of a sibling, sibling incest or abuse, or sibling intervention or psychometric testing; non-English articles; and those without full text. The data extraction and analysis involved coding the study characteristics and results related to sibling relationship quality and providing a comprehensive overview of the research landscape. 

For this paper, we utilized the 277 articles identified in the EGM by applying additional inclusion criteria to focus specifically on Latinx populations. To be included in this systematic review, studies had a United States sample of 50% or more Latinx participants. Following the completion of the independent screening process by two researchers, we conducted a thorough review of the full-text articles. One researcher extracted information related to the study design, sampling methods, cultural factors present and measured, and associations between cultural factors and sibling relationship dynamics. Data on domains of sibling relationship quality, methodological approaches, and variables associated with positive sibling relationships were systematically entered into a data extraction form.

## 3. Results

A total of 12 articles were identified from the Holmes et al. [9] EGM as having a United States sample consisting of 50% or more Latinx participants (see Figure 1).

### 3.1. Characteristics of Included Studies

Table 1 provides a detailed description of the 12 included articles. The included articles were published between 1997 and 2021. Most of the included articles (*n* = 9), across five studies, utilized longitudinal study designs, while three articles, across two studies, utilized cross-sectional study designs. Most articles utilized Mexican-American samples (92%, *n* = 11); the remaining study did not specify their sample’s ethnicity, noting that most questions were conducted in Spanish [39]. Family sample sizes ranged from 55 [40] to 404 [41]. Among the 12 included articles, only 6 represent distinct studies with distinct samples. Notably, two of six studies generated multiple publications: Updegraff et al.’s [29] study contributed five articles, while Modry-Mandell et al.’s [42] study produced three articles. In this review, the term ‘study’ refers to original research that collected primary data, while ‘article’ denotes publications that reported secondary analyses or additional insights based on the data from these studies. Thus, although we included 12 articles, they collectively represented findings from 6 distinct studies.

Table 2 presents the aggregated study sample descriptives of the six distinct studies. The majority of the studies (*n* = 4; 67.67%) did not report whether the households consisted of two caregivers, while two studies (33.33%) required two-caregiver households as a criterion for participation. Of those studies that reported family income (*n* = 4; 66.67%), none reported a majority upper- or middle-class income. The average age of the index sibling was 12.50 years (SD = 5.37), ranging from 4.79 to 17.26 years. When both ages of the siblings were reported, the average age of the youngest sibling in the dyad was 13.34 years (SD = 0.80), while the oldest sibling averaged 16.60 years (SD = 1.27). Only one study (16.67%) reported including siblings other than full siblings.

### 3.2. Sibling Relationship Quality Measurement

Table 3 provides aggregated descriptive information on the methodologies and measures used to assess sibling relationship quality by study sample. All six studies (100%) utilized quantitative measures. For reporting on sibling relationship quality, most studies (*n* = 5; 83.33%) relied on reports from children only, while one study (16.67%) used caregiver reports. No studies used both caregiver and child reports. Of the studies that utilized child reports, only one (20%) included reports from two siblings. The most commonly used quantitative measure across the studies was the Sibling Intimacy Scale [54], used in five articles (41.67%), all from the Updegraff et al.’s [29] study. The Network of Relationships Inventory [53] and the Parental Expectations and Perceptions of Children’s Sibling Relationship Quality Questionnaire [49] were each used in three articles (25%). In contrast, the Sibling Relationship Questionnaire [48] was used in two articles (16.67%).

**Figure 2:**
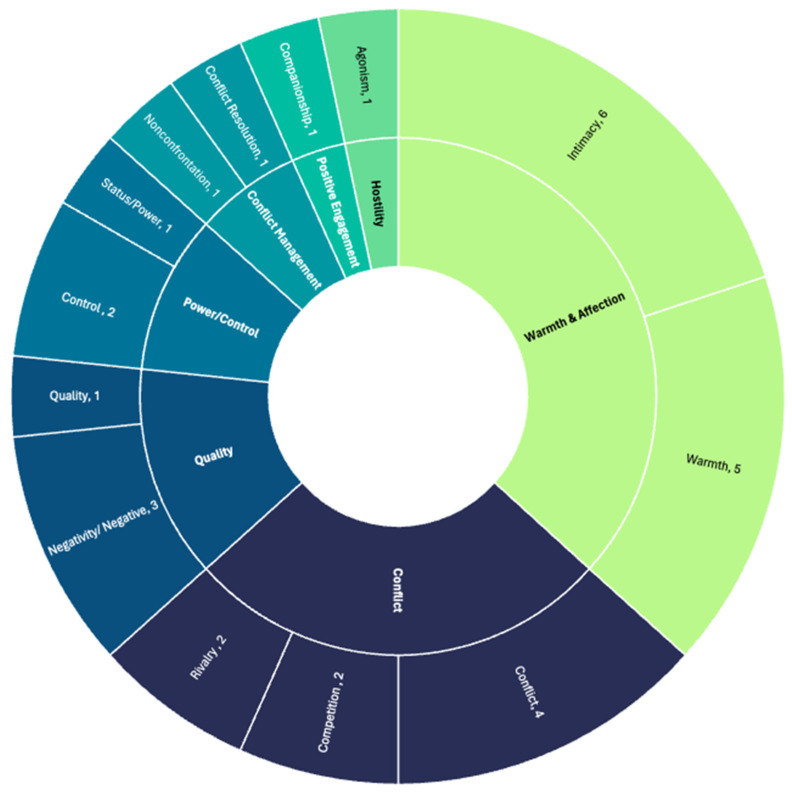
Domains and the constructs of sibling relationship quality measured showing the relative sizes of domains (inner circle) and constructs (outer circle).

Figure 2 displays the domains and the constructs of sibling relationship quality measured in the included articles, which visualizes the relative sizes of domains (inner circle) and constructs (outer circle), where larger spaces mean that the domain or construct was measured more often. The two most frequent domains measured were warmth, with constructs of intimacy (*n* = 6) and warmth (*n* = 5), and conflict, with constructs of conflict (*n* = 4), competition (*n* = 2), and rivalry (*n* = 2). Positive engagement and hostility were the least frequent domains of sibling relationship quality measured. Quality refers to general quality (i.e., high vs. low quality or positive vs. negative quality). No constructs of cohesion (e.g., support, closeness, and perspective-taking) were measured across articles.

### 3.3. Cultural Factors

The cultural factors identified in these articles largely consisted of family cultural values (*n* = 7), such as familism and simpatía; the nativity status of the respondents, parents, or family members (*n* = 5); and cultural orientation (*n* = 1). Notably, three articles did not include cultural factors in their analyses [39,42,47]. The most commonly studied cultural factor, the family cultural value of familism, was examined through two measures: Family Relationships Values Q-Sort (*n* = 2) [50] and the Familism subscale from the Mexican American Cultural Values Scale (*n* = 5) [46]. The Familism subscale from the Mexican American Cultural Values Scale included three domains: support/closeness, family obligations, and family as a referent [46]. Simpatía was also examined in two articles, which utilized the scale developed by Griffiths et al. [51]. Nativity was examined through three aspects: country of origin (*n* = 2), immigration status (*n* = 2), and number of years in the U.S. (*n* = 4). One article [49] included a cultural orientation measure (ARSMA-II) [60], which consists of two subscales to assess an individual’s orientation to Anglo and Mexican culture.

Gender constellation was also examined in eight articles (67%) [24,29,40,41,43,56,57,58]. Typically, gender dyads were reduced to same- and opposite-sex dyads for analyses (*n* = 5). The included articles also revealed some gender-related findings. Alfaro and Umaña-Taylor [43] found that sibling relationship quality was directly related to girls’ academic motivation, indicating the significant impact of positive sibling interactions on academic outcomes for female siblings. Cruz et al. [41] found that familism moderated the relationship between sibling intimacy and later alcohol use patterns. In families with lower levels of familism, increased intimacy between siblings was linked to a higher likelihood of any alcohol use but a decrease in the extent of use, particularly among brother and sister pairs. Conversely, in families with higher levels of familism, greater sibling intimacy was associated with a lower probability of any alcohol use but an increase in the degree of use, especially for sister pairs and mixed-gender siblings. Killoren et al. [57] highlighted that older sibling gender moderated the link between familism in middle adolescence and sibling intimacy in young adulthood, with mixed-gender dyads showing different patterns from girl–girl dyads, indicating that gender composition influences how cultural values affect sibling intimacy over time. In contrast, Killoren et al. [58] found that sibling gender and the gender constellation of the sibling dyad were not significant correlates of conflict resolution strategies.

### 3.4. Influence of Cultural Factors on Sibling Relationship Quality

Several articles explored familism’s association with sibling relationship qualities, including intimacy, warmth, closeness, positive domains, negative domains, and siblings’ solution orientation. The intimacy domain was the most thoroughly explored, where familism was associated with increased sibling intimacy (*n* = 4) [29,41,56,57]. One article determined that stronger familism in adolescents and sibling intimacy predicted positive values in later adolescence and decreased risky and sexual risk behaviors [56]. Additionally, Killoren et al. [56] found that where strong familism exists, sibling intimacy predicted decreases in depressive symptoms. Killoren et al. [57] found that older sibling gender moderated the link between familism in middle adolescence and sibling intimacy in early adulthood. Two articles found that familism was associated with siblings’ closeness [29] and higher levels of warmth [52]. Familism values were positively associated with siblings’ solution orientation [58]. Two articles found no significant relationship between familism and negativity [41] or positive sibling relationship profiles [24]. However, Killoren et al. [24] conducted post hoc tests that showed positive sibling relationship profiles reported higher familism than affect-intense and negative sibling relationship profiles.

Two articles found that simpatía was associated with higher levels of sibling warmth [40,52]. While there was some evidence for a relationship between nativity and sibling relationship quality, it was not particularly strong. One article found a significant negative correlation between being born in the U.S. and sibling relationship quality [43]. Additionally, Updegraff and colleagues [29] reported that siblings whose fathers were born in Mexico, and who spent more time with parents born in Mexico, reported less conflict. However, these effects were minor [29]. Both Anglo and Mexican cultural orientations were positively linked to siblings’ solution orientation strategies [58].

### 3.5. Methodological Considerations 

The most common theoretical perspective in these articles was social learning theory (50%, *n* = 6) [61]. The second most common theoretical perspective was Bronfenbrenner’s [62] ecological model (42%, *n* = 5). One article drew directly from Bronfenbrenner, whereas other authors sought more contextual ecological models; for example, Gamble and Modry-Mandell [40] drew on a familiar ecological context. Three articles (25%) utilized a cultural–ecological perspective [63,64]. Two articles (17%) [52,57] drew on family systems theory, primarily citing Minuchin [65], Minuchin [66], and Cox and Paley [67]. Notably, these articles both paired family systems theory with social learning theory. One article (8%) engaged the risk and resilience perspective [68] along with social learning theory. Two articles did not note a guiding theoretical perspective (17%) [41,42].

Across the included articles, 83% (*n* = 5) included an interview component, and 67% included a questionnaire (*n* = 4). Nearly all of the articles offered bilingual data collection (92%, *n* = 11). The single article that did not offer bilingual data collection was conducted among students in midwestern schools [43].

A variety of analytical techniques were used. Half of the included articles (*n* = 6) utilized some regression analyses, including general regression analyses (*n* = 2), hierarchical regression (*n* = 2), logistic regression (*n* = 1), and Poisson regression (*n* = 1). Several structural equation modeling (SEM) techniques were also employed by 41% of the included studies (*n* = 5): general SEM (*n* = 2), latent profile analysis (LPA; *n* = 1), multi-level modeling (MLM; *n* = 1), and path analyses (*n* = 1). One-third of the articles utilized ANOVA analyses: mixed-model ANOVA (*n* = 2) and mixed-model ANCOVA (*n* = 2). One article used a cluster analysis. Most articles included did not require the use of nested models (75%, *n* = 9) due to their reliance on only one sibling’s report, or averaged reports, for the sibling relationship quality measure. One article did not use a nested model [56], and two articles included a nested model for structural equation modeling and multi-level modeling [57,58].

## 4. Discussion

The present systematic review aimed to synthesize the existing research on sibling relationship quality among Latinx families, focusing on cultural influences, associated factors, and methodological approaches. Our analysis included 12 articles representing 6 distinct studies, revealing several critical insights into sibling dynamics within Latinx families. This review fills a critical gap in the literature by providing a comprehensive synthesis of the unique cultural factors influencing sibling relationships in Latinx families while also highlighting the need for future research to explore these dynamics further and to expand the scope to include a more diverse range of sibling and family experiences.

### 4.1. Sibling Relationship Quality Measurement 

While the included articles measured several important constructs of sibling relationship quality such as warmth, conflict, power/control, and conflict management, it was evident that certain key constructs were also missing. For example, prosocial domains were notably absent when comparing the 12 included articles to the broader sibling relationship quality research [9]. Prosocial behaviors, such as helping, sharing, and comforting, are crucial for fostering strong supporting sibling relationships [69,70]. These behaviors have been found to contribute to the overall quality of the sibling relationship as well as multiple social outcomes [71]. The absence of measures for prosocial behaviors limits the ability to fully understand the positive aspects of sibling interactions, which are particularly important in the context of Latinx families, where cohesion and mutual support are highly valued. 

Many of the measures used in the included articles were not developed with diverse socioeconomic samples but rather used primarily white, middle-to-upper class, two-parent intact families [48,49,53,54]. There was only one measure that was developed or tested in a racially and ethnically diverse sample. Thayer et al.’s [59] measure focuses on conflict resolution in Mexican-American adolescents’ friendships, exploring the role of cultural orientation and values, as well as gender-typed personality qualities in conflict resolution use. Although not specifically designed for sibling relationships, this measure was adapted to reflect cultural nuances relevant to Mexican-American families. It may be that other cultural factors, such as the concepts of machismo and marianismo, also play a role in shaping sibling dynamics. Still, these have not been explicitly examined in the included studies. Developing and testing measures that account for these and other culturally specific factors will be crucial for future research.

The most common measure used in the included articles was the Sibling Intimacy Scale [54], which was originally developed to assess intimacy in close relationships among adolescents including peers and adults (e.g., parents and teachers). While this measure can provide some insights, it may not fully capture the unique aspects of sibling intimacy. Intimacy in sibling relationships may differ from close relationships with peers and adults because it includes elements such as shared experiences, mutual support, and familial bonding. Using a measure designed for peer and adult close relationships could lead to incomplete or inaccurate understandings of sibling intimacy. 

### 4.2. Cultural Factors

Our review identified familism and simpatía as key cultural factors influencing sibling relationship quality [72]. Familism, a core value in Latinx cultures, was consistently associated with positive sibling qualities, including increased intimacy, warmth, and solution orientation [29,41,52,56,57,58]. Notably, articles found that higher familism values were linked to reduced depressive symptoms and risky behaviors, emphasizing the protective role of cultural values [56]. Simpatía was also associated with higher levels of sibling warmth, further underscoring the influence of cultural values on sibling dynamics [40,52]. 

The influence of nativity on sibling relationship quality was less pronounced. While some articles reported minor associations between nativity and sibling dynamics, these effects were not consistently strong [29,43]. Additionally, cultural orientation, measured through both Anglo and Mexican cultural perspectives, was positively linked to sibling solution orientation strategies, suggesting that bicultural identities may foster adaptive conflict resolution [58].

This review’s findings reinforce sibling relationships’ central role in Latinx family life. Familism and simpatía are cultural factors relevant to Latinxs that value family cohesion and support, contributing to stronger sibling bonds and overall family stability. These cultural values promote positive interactions and less conflict, creating a nurturing environment for child development [40,52,56]. Positive sibling interactions provide emotional support and buffer against external stressors, promoting healthy development [73]. The protective effects of sibling warmth and intimacy, coupled with the influence of familism, underscore the importance of nurturing these relationships to enhance youth development [29,56]. This review also highlights the importance of maintaining cultural values like familism and simpatía in the process of cultural adaptation. These values not only strengthen family ties but also support resilience in the face of acculturative stress. By fostering a sense of belonging and mutual support, these cultural ideals help Latinx families navigate the challenges of adapting to a new cultural context [56,57].

While the included articles have examined the roles of familism and simpatía in influencing sibling relationship quality among Latinx families, there are several other important cultural factors that merit attention. Machismo and marianismo, which define traditional male and female gender roles, respectively, can significantly shape sibling dynamics by reinforcing gender-specific behaviors and expectations [33]. The value of respect/respeto, emphasizing respect for authority and social hierarchy, also plays a crucial role in family interactions, potentially affecting sibling relationships through the enforcement of hierarchical norms [74]. In the context of Latinx Americans, acculturation and enculturation processes are also vital [75], as they impact how siblings adapt to and maintain cultural norms, potentially affecting their interactions and cohesion. The integration of bicultural identity, where individuals balance aspects of both Latinx and mainstream American cultures [76], might influence how siblings navigate and reconcile different cultural expectations. Siblings are critical role models and sources of support as children and adolescents crystallize their understanding of what it means to be a person of their culture and ethnicity [77], as well as navigate experiences of discrimination and racism [78]. Another culturally salient family process that may shape the quality of sibling relationships is sibling caregiving [79]. Margolis et al. [80] found that 35% of U.S. Latinx adolescents considered their older siblings to be caregivers, in contrast to 17% of African-American and 10% of European-American adolescents. Sibling caregiving reflects and promotes children’s socioemotional development, building empathy, perspective-taking, and self-reliance [79]. In some cases, sibling caregiving can be a family survival strategy; for example, in immigrant families, sibling caregiving can extend to being a translator, culture broker, and advocate for the entire family. Understanding these cultural factors alongside familism and simpatía can provide a more comprehensive picture of sibling relationship quality in Latinx families, highlighting the complex interplay of cultural values in shaping family dynamics and child social–emotional outcomes.

Gender constellation and familism were shown to have a significant association with sibling intimacy in 25% (*n* = 3) of articles among sister dyads [29,41,57] and mixed pairs [41]. Updegraff and colleagues [29] also found that familism was associated with closeness, particularly among sisters. Two articles found gender constellation to be significant when considering risky sexual behavior [24,56]. When operating as a control variable, gender constellation significantly predicted risky sexual behavior [56], and for positive and negative sibling profiles, younger siblings reported higher levels of risky sexual behavior than their older siblings [24]. One article assessing academic motivation found that sibling academic support was positively associated with academic motivation among boys but not girls [43]. However, sibling relationship quality was positively related to girls’ academic motivation, and nativity was negatively related to girls’ academic motivation [43]. These findings underscore the importance of considering gender roles and expectations within the cultural context of Latinx families. Future research should further explore how gender roles evolve over time and across different developmental stages, as well as how they interact with other cultural factors such as familism. Understanding these dynamics can inform the development of culturally sensitive interventions that promote positive sibling relationships and support healthy development for both boys and girls in Latinx families. 

Despite previous findings, contextual factors such as socioeconomic status and experiences of discrimination were not given a strong role in this literature [81,82,83,84]. Socioeconomic status was most often conceptualized as annual income *(n* = 7) and parental education (*n* = 8); however, most articles included one or both measures separately in their models. Only Killoren et al. [57] included a composite socioeconomic status score derived from annual income and the mother and father’s education. Two articles included no information on socioeconomic status, but both had entirely low-income samples [42,52]. Experiences of discrimination or systematic disadvantage were mentioned in two articles [29,52].

### 4.3. Methodological Considerations 

Theoretical frameworks provide essential lenses through which sibling relationship quality can be understood, particularly within the cultural context of Latinx families. Mainstream theories, such as social learning theory [61] and Bronfenbrenner’s [62] ecological model, were most often used in the included articles, yet only three articles utilized a cultural–ecological perspective, integrating cultural and ecological factors to explore how cultural norms and values shape sibling dynamics [63,64]. While these theories have provided valuable insights, other theories may also be relevant for studying sibling relationship quality among Latinx families. For example, acculturation theory [76] could offer insights into how siblings navigate the integration of Latinx and American cultural values. Intersectionality theory [85] could elucidate how overlapping social identities, such as race, ethnicity, gender, and socioeconomic status, influence sibling relationships. Integrating such theories could enhance the understanding of sibling relationship quality and inform future culturally sensitive interventions.

Most articles in this review collected data about adolescent sibling relationships, with only one study collecting information about preschool-aged siblings. None of the articles were completed with siblings between the ages of 6 and 12. Future studies should include a wider age range to account for differences in sibling relationships throughout childhood. Research has shown that sibling relationships evolve significantly across different developmental stages. In early childhood, sibling interactions often revolve around play and learning social norms, with older siblings sometimes taking on caregiving roles [86]. Middle childhood is marked by increased cognitive and social development, leading to more complex interactions and the potential for both conflict and cooperation [87]. Adolescence, a period of significant individual identity formation and increased autonomy, often sees shifts in sibling dynamics as relationships may become more egalitarian or, alternatively, more strained due to increased individuation or peer influence [2]. Examining sibling relationships across these stages is crucial to fully understand the developmental trajectory and the factors that influence sibling bonding. This is especially important in the context of Latinx families, where cultural values such as familism emphasize strong family bonds and mutual support. By including a broader age range in future studies, researchers can capture the nuances and variations in sibling relationships, providing a more comprehensive understanding of how these relationships develop and change throughout childhood and adolescence.

Latinx families tend to have larger family sizes, yet little research has examined the relationships of more than two siblings [9]. Livingston [88] reported that 50% of Latina women had three children or more, which exceeded that of White (33%), Black (40%), and Asian (27%) mothers. With opportunities to interact with more siblings, Latinx children and adolescents may reap more of the benefits (and challenges) that come with these relationships, yet due to methodological limitations, we have not fully studied these complex sibling relationships. Furthermore, the included articles studied sibling relationships between fully biological sibling dyads in two-parent households. Our understanding of family dynamics, as well as cultural influences on them, has evolved considerably. Recent research indicates that the complexity of family structures, such as blended and single-parent households, has gained more recognition in recent years, prompting the need for updated frameworks to conceptualize family and sibling relationships. Further, important work has expanded knowledge about how family is culturally defined [89,90]. In the future, it is important to investigate sibling relationships between half-siblings, step-siblings, and adoptive siblings, in addition to single-parent and blended family households. The prevalence of longitudinal study designs among the included articles is a notable strength, allowing for the examination of sibling dynamics over time. Longitudinal approaches provide critical insights into the developmental trajectories of sibling relationships and the long-term impact of cultural factors. The utilization of multiple data collection methods is an additional strength. The studies gathered data through sibling reports, maternal reports, paternal reports, teacher reports, and several other questionnaires and interviews. In this way, the study captured the perspectives of multiple individuals, ultimately providing a deeper understanding of sibling relationships inside and outside the home environment. However, none of the included articles used qualitative methods; future research should prioritize the inclusion of qualitative studies to achieve a more comprehensive understanding. Qualitative methods can offer deeper insights into the lived experiences and personal narratives of siblings, revealing the nuanced ways cultural factors such as familism, simpatía, and gender roles shape their interactions and relationships. By capturing the voices and perspectives of Latinx siblings directly, qualitative research can uncover the rich, contextualized details that quantitative measures may overlook.

The 12 articles draw from 6 studies, which speaks to a need for more diverse samples and accounts for the near-universal use of Mexican-American samples. This review highlights the need for more research on sibling relationships in diverse Latinx subgroups beyond Mexican-Americans. For example, Puerto Rican-Americans and Salvadorans are the two Latinx subgroups with the highest populations in the United States beyond Mexican-Americans. As of 2021, approximately 5.8 million Puerto Rican-Americans and 2.5 million Salvadorans resided in the United States [91]. Future studies should include a broader range of Latinx populations to capture the heterogeneity within this group. 

### 4.4. Intervention and Policy Implications

Few prevention or intervention programs have been developed to strengthen sibling relationships in Latinx families. One exception is the Siblings are Special (SIBS) program [92], a 12-session after-school program for 5th graders and a younger sibling that was designed to be applicable for European-American, African-American, and Latinx families. Updegraff et al. [26] evaluated a modified version of SIBS with 54 low-income Latinx families of predominantly Mexican descent and found greater prosocial and fewer negative sibling behaviors at the post-test in comparison to a randomly assigned control group. Updegraff et al. attributed the families’ satisfaction with the program to its alignment with Latino cultural values, e.g., fostering close and supportive family relationships. However, cultural values, such as familism and simpatía, were not directly assessed. Thus, we have more to learn about how Latinx values and parenting practices promote prosocial sibling relationships. Additional programs that are designed with Latinx populations, that incorporate key cultural values and practices, and that serve siblings of different developmental levels are sorely needed. Program participation and sustained application of its principles to the home context are more likely to occur if the program adheres to the values and practices that Latinx families are most committed to. 

We encourage future research to explore the sibling interpersonal processes highly valued in Latinx families, including familism, simpatía, and cohesion. As we better understand the factors that predict prosocial sibling relationships in Latinx families, we will be better able to design effective culturally sensitive tools for prevention and intervention. Similarly, greater knowledge of how Latinx parents transmit their core cultural values to their children in ways that promote strong sibling relationships will better inform the design of culturally responsive interventions. Given the encouraging results we reported in this review, in which the transmission of Latinx values is linked with prosocial sibling relationships, we expect that prevention and intervention initiatives that incorporate the voice of Latinx families into their design will produce the most pronounced and sustained effects on children’s and adolescents’ sibling relationships. 

### 4.5. Strengths and Limitations

This review is comprehensive in scope, synthesizing a diverse body of literature on sibling relationship quality among Latinx families. The inclusion of various methodological approaches and the focus on cultural factors provide a foundation for understanding sibling dynamics in this context. However, several limitations should be noted. First, this study drew the included studies from the Holmes et al. [9] EGM, which means we did not conduct an independent search specifically for Latinx sibling research. Instead, we utilized the robust data extracted from the 277 studies included in the EGM to identify our sample of studies. This reliance on the EGM may have inadvertently excluded relevant articles if they were not part of the original Holmes et al. [9] compilation. Non-English articles were excluded from this review, which may have resulted in the omission of studies published in Spanish that explore sibling relationship quality among Latinx families. This language limitation could potentially bias the results toward English-language research, neglecting valuable insights from non-English sources. Additionally, studies that focused solely on the parenting practices of siblings were excluded; only studies that directly examined sibling relationship quality were included in this review. This exclusion is a limitation because parenting practices can significantly influence sibling dynamics and excluding such studies may result in a less comprehensive understanding of the factors that shape sibling relationships within Latinx families. Finally, this review focused specifically on Latinx sibling relationships, providing an in-depth understanding of this particular cultural context. However, due to this narrow cultural focus, the findings may not be generalizable to sibling relationships in other cultural groups. Future research should aim to investigate sibling relationships across various cultures in the United States to develop a more comprehensive understanding of how cultural factors influence sibling dynamics. 

## 5. Conclusions

In summary, this systematic review contributes to a deeper understanding of sibling relationship quality in Latinx families, highlighting the influence of cultural values, methodological considerations, and the implications for family dynamics, cultural adaptation, and resilience. The findings underscore the importance of culturally responsive approaches in research, practice, and policy to support the well-being of Latinx families. Future research should continue to explore the complexities of sibling relationships in diverse cultural contexts, advancing our knowledge and informing effective interventions.

## Figures and Tables

**Table 1:** Study descriptives of sibling relationship quality and cultural factors among Latinx families (*n* = 12).

Citation	Design & Sample Characteristics	Sibling Relationship Quality Measurement	Cultural Factors	Significant Cultural Factors Findings	Other Significant Findings
Alfaro & Umaña-Taylor (2010) [43]	Longitudinal Families (*n* = 258) 77.1% Mexican-American (siblings) Index sibling age *M* = 17.26 y Two-parent household NR	Child report Quantitative Sibling relationship quality and the culture of openness and disclosure [44]; quality	Nativity Gender	Being born in the U.S. was negatively correlated with sibling relationship quality. Sibling’s academic support was positively associated with academic motivation for boys but not girls. Sibling relationship quality was positively related to girls’ academic motivation. Nativity negatively related to girls’ academic motivation.	Sibling relationship quality was positively associated with sibling academic support and academic motivation.
Cruz et al. (2019) [41]	Longitudinal Families (*n* = 404) 100% Mexican-American (siblings) Index sibling age *M* = 14.26 y Two-parent household NR	Child report Quantitative Sibling Closeness Scale [45]; intimacy, negative	Mexican American Cultural Values Scale [46]; familism (composite score of family support, emotional closeness, family referent, and family obligations) Gender	Familism was related to higher sibling intimacy in sibling relationships. Familism was related to lower alcohol use. Familism moderated the effects of sibling intimacy on later alcohol use. Lower familism and increasing intimacy were associated with a higher probability of any use. Higher familism increasing intimacy reduced the probability of use but increased the degree of use for sisters and mixed pairs.	Age 14 sibling negativity was associated with alcohol use. Sibling negativity was related to reduced alcohol use probability for brothers and increased alcohol use in mixed sibling pairs.
East & Shi (1997) [47]	Cross-sectional Families (*n* = 80) 68% Mexican-American (siblings) Youngest sibling age *M* = 13.90 y Oldest sibling age *M* = 17.5 y Two-parent household NR	Child report Quantitative Sibling Relationship Questionnaire [48]; conflict, rivalry, status/power, warmth	None	NA	School/career orientation was positively associated with sibling warmth. Problem behaviors were positively associated with rivalry/parent partiality and conflict. Sexual permissiveness and sexual status were positively associated with sibling rivalry. Negative sibling relationship qualities (rivalry, competition, and conflict) were more closely related to younger sisters engaging in problem, delinquent-like behavior and sexual behavior than positive relationships.
East et al. (2007) [39]	Longitudinal Families (*n* = 127) 57% Mexican-American (mothers) Index sibling age *M* = 13.7 y Two-parent household NR	Child report Quantitative Sibling Relationship Questionnaire [48]; conflict, rivalry, warmth, companionship	None	NA	Compared with young women with no family history of teenage births, young women whose sister had had a teenage birth and those whose sister and mother both had had teenage births were significantly more likely to experience a teenage pregnancy. Having both a mother and a sister who had had teenage births was independently associated with an elevated risk of pregnancy, even after controlling for socioeconomic and mothers’ parenting characteristics.
Gamble & Modry-Mandell (2008) [40]	Longitudinal Families (*n* = 55) 100% Mexican-American (mothers) Index sibling age *M* = 4.79 y Two-parent household 100%	Parent report Quantitative Parental Expectations and Perceptions of Childrens Sibling Relationship Quality Questionnaire [49]; warmth	Relational Family Values Q-sort [50]; familism Simpatía Scale [51]; simpatía Gender	Simpatia was positively correlated with sibling warmth. Sibling warmth was significantly positively correlated with simpatia, mother–child closeness, emotional adjustment, and peer adjustment. Familism has a direct effect on predicting externalizing behaviors, controlling for sibling warmth. Familism and mother–child closeness predicted children’s emotional adjustment. Interaction between familism and sibling warmth predicted children’s emotional adjustment and peer adjustment. No significant gender differences were found for familism, simpatía, or sibling warmth.	Sibling warmth was significantly negatively correlated with internalizing and externalizing behaviors. Sibling warmth had a direct effect on behavior problems. Sibling warmth predicted peer adjustment.
Gamble & Yu (2014) [52]	Longitudinal Families (*n* = 65) 95% Mexican-American (mothers) Index sibling age *M* = 4.79 y Two-parent household 100%	Parent report Quantitative Parental Expectations and Perceptions of Childrens’ Sibling Relationship Quality Questionnaire [49]; conflict, competition, warmth,	Relational Family Values Q-sort [50]; familism Simpatía Scale [51]; simpatía	Mothers’ higher simpatia and familism scores were associated with higher levels of sibling warmth.	Families characterized by more positive emotions were more likely to have children in sibling relations characterized by high levels of warmth and low levels of conflict.
Killoren et al. (2017) [24]	Longitudinal Families (*n* = 246) 100% Mexican-American (siblings) Youngest sibling age *M* = 12.77 y Oldest sibling age *M* = 15.7 y Two-parent household 100%	Child report Quantitative Network of Relationships Inventory [53]; negative quality Sibling Intimacy Scale [54]; intimacy Perceived sibling control [55]; control	Years living in the United States Mexican American Cultural Values Scale [46]; familism (support/closeness, family obligations, and family as referent) Gender	Siblings with a positive relationship profile reported higher familism than siblings in the affect-intense and negative profiles. Older sibling gender moderated the link between familism in middle adolescence and sibling intimacy in young adulthood. For positive and negative profiles, younger siblings reported significantly higher levels of risky behaviors than their older opposite-sex siblings.	T1 depressive symptoms were a significant positive covariate, and there was a significant profile X birth order interaction such that profile differences in depressive symptoms emerged for older, but not younger, siblings. Older siblings in the negative profile reported higher depressive symptoms than older siblings in the positive and affect-intense profiles. For the negative profile, younger siblings reported higher levels of sexual risk behaviors than older siblings at T2, but there were no significant differences between older and younger siblings’ sexual risk behaviors at T3.
Killoren et al. (2021) [56]	Longitudinal Families (*n* = 246) 100% Mexican-American (siblings) Youngest sibling age *M* = 12.77 y Oldest sibling age *M* = 15.7 y Two-parent household 100%	Child report Quantitative Sibling Intimacy Scale [54]; intimacy	Mexican American Cultural Values Scale [46]; familism (support/closeness, family obligations, and family as referent) Gender	Under conditions of stronger familism values, sibling intimacy in early adolescence predicted more positive values in later adolescence, which, in turn, led to relatively lower levels of risky behaviors and lower sexual risk behaviors in young adulthood. Sibling intimacy in early adolescence predicted younger siblings’ adjustment problems in young adulthood via their positive values in later adolescence, but only for younger siblings with strong familism values.	NA
Killoren et al. (2015)[57]	Longitudinal Families (*n* = 246) 100% Mexican-American (siblings) Youngest sibling age *M* = 12.55 y Oldest sibling age *M* = 15.49 y Two-parent household 100%	Child report Quantitative Sibling Intimacy Scale [54]; intimacy	Nativity status of all family members; Years living in the United States; Mexican American Cultural Values Scale [46]; familism (support/closeness, family obligations, and family as referent) Gender	Familism values were associated with increased sibling intimacy during young adulthood Older sibling gender also moderated the link between familism in middle adolescence and sibling intimacy in young adulthood. Gender constellation moderated the link between youth’s familism in middle adolescence and sibling intimacy during young adulthood such that the mixed-gender dyads were significantly different from the girl–girl dyads.	NA
Killoren et al. (2008) [58]	Cross-sectional Families (*n* = 246) 100% Mexican-American (siblings) Youngest sibling age *M* = 12.8 y Oldest sibling age *M* = 15.7 y Two-parent household 100%	Child report Quantitative Network of Relationships Inventory [53]; conflict Sibling Intimacy Scale [54]; intimacy Resolving Conflict in Relationship Scale [59]; control, solution orientation, non-confrontation	Nativity; years living in the United States; ARSMA-II [60]; cultural orientations (individual’s orientation to Mexican and Anglo culture) Gender	Cultural orientations and familism values were positively linked to siblings’ solution orientation. Actor familism and partner Mexican orientation were positively related to using non-confrontational strategies between siblings, whereas partner Anglo orientation was negatively related to non-confrontation. For solution orientation, there were significant positive effects for actor Mexican orientation, actor Anglo orientation, and actor and partner familism. The controlling model revealed that actor Anglo cultural orientation was positively related to controlling strategies. Bicultural-oriented adolescents (i.e., adolescents who fell above the median on Anglo and Mexican orientations) significantly used solution orientation more than adolescents who were not bicultural. Sibling gender and the gender constellation of the sibling dyad were not significant correlates of resolution strategies.	NA
Modry-Mandell et al. (2007) [42]	Longitudinal Families (*n* = 55) 95% Mexican-American (mothers) Index sibling age *M* = 4.79 y Two-parent household 96%	Parent report Quantitative Parental Expectations and Perceptions of Children’s Sibling Relationship Quality Questionnaire [49]; competition, agonism, warmth	None	NA	Sibling warmth negatively predicted children’s behavior problems and positively predicted children’s adaptation.
Updegraff et al. (2005) [29]	Cross-sectional Families (*n* = 246) 100% Mexican-American (siblings) Youngest sibling age *M* = 12.8 y Oldest sibling age *M* = 15.7 y Two-parent household 100%	Child report Quantitative Network of Relationships Inventory [53]; negative quality Sibling Intimacy Scale [54]; intimacy	Nativity; Years living in the United States; Mexican American Cultural Values Scale [46]; familism (support/closeness, family obligations, and family as referent) Gender	Sibling pairs spent more time together when parents were born in Mexico and reported less conflict when fathers were born in Mexico, but the effects were small. Cultural background characteristics were not related to adolescents’ familistic values and practices. Familism was associated with siblings’ feelings of intimacy and closeness and showed some links with siblings’ dyadic time, especially for sisters.	NA

**Table 2:** Study sample descriptives of distinct studies (*n* = 6).

	*n*/*M* (SD)	%/Range
Two-caregiver households		
100%	2	33.33%
Not reported	4	67.67%
Family income majority upper or middle class		
Yes	0	0.00%
No	4	66.67%
Not reported	2	33.33%
Age of sibling (years)		
Age of index sibling	12.50 (5.37)	4.79–17.26
Age of youngest sibling in dyad	13.34 (0.80)	12.77–13.9
Age of oldest sibling in dyad	16.60 (1.27)	15.7–17.5
Siblings other than full included		
Yes	1	16.67%
No (full only)	1	16.67%
Not reported	4	66.67%

**Table 3:** Sibling relationship quality study descriptives (*n* = 6).

	*n*/*M* (SD)	%/Range
Methodology used in measure		
Quantitative measure	6	100%
Sibling relationship quality reporter of quantitative measures		
Caregiver only	1	16.67%
Child only	5	83.33%
Both caregiver and child	0	0.00%
Number of children who reported sibling relationship quality (*n* = 5)		
1 sibling	4	80.00%
2 siblings	1	20.00%
Most commonly used quantitative measures ^1^		
Sibling Intimacy Scale [54]	5	41.67%
Network of Relationships Inventory [53]	3	25.00%
Parental Expectations and Perceptions of Children’s Sibling Relationship Quality Questionnaire [49]	3	25.00%
Sibling Relationship Questionnaire [48]	2	16.67%

^1^ Descriptive information based on articles (*n* = 12).

## Data Availability

No new data were created or analyzed in this study. Data sharing is not applicable to this article. This review and the protocol were not registered.
